# COVID-19 illness in relation to sleep and burnout

**DOI:** 10.1136/bmjnph-2021-000228

**Published:** 2021-03-22

**Authors:** Hyunju Kim, Sheila Hegde, Christine LaFiura, Madhunika Raghavan, Eric Luong, Susan Cheng, Casey M Rebholz, Sara B Seidelmann

**Affiliations:** 1 Department of Epidemiology, Johns Hopkins Bloomberg School of Public Health, Baltimore, Maryland, USA; 2 Welch Center for Prevention, Epidemiology and Clinical Research, Johns Hopkins University, Baltimore, MD, USA; 3 Division of Cardiovascular Medicine, Brigham and Women’s Hospital, Boston, Massachusetts, USA; 4 Harvard Medical School, Boston, MA, USA; 5 Envision Health Partners, Stamford, CT, USA; 6 Department of Cardiology, Schmidt Heart Institute, Cedars-Sinai Medical Center, Los Angeles, CA, USA; 7 Department of Medicine, Stamford Hospital, Stamford, Connecticut, USA; 8 Department of Medicine, Columbia University College of Physicians & Surgeons, New York, NY, USA

**Keywords:** COVID-19, sleep

## Abstract

**Background:**

Sleep habits and burnout have been shown to be associated with increase in infectious diseases, but it is unknown if these factors are associated with risk of COVID-19. We assessed whether sleep and self-reported burnout may be risk factors for COVID-19 among high-risk healthcare workers (HCWs).

**Methods:**

From 17 July to 25 September 2020, a web-based survey was administered to HCWs in six countries (France, Germany, Italy, Spain, UK, USA) with a high frequency of workplace exposure. Participants provided information on demographics, sleep (number of sleep hours at night, daytime napping hours, sleep problems), burnout from work and COVID-19 exposures. We used multivariable linear and logistic regression models to evaluate the associations between sleep, burnout and COVID-19.

**Results:**

Among 2884 exposed HCWs, there were 568 COVID-19 cases and 2316 controls. After adjusting for confounders, 1-hour longer sleep duration at night was associated with 12% lower odds of COVID-19 (p=0.003). Daytime napping hours was associated with 6% higher odds, but the association varied by countries, with a non-significant inverse association in Spain. Compared with having no sleep problems, having three sleep problems was associated with 88% greater odds of COVID-19. Reporting burnout ‘every day’ was associated with greater odds of COVID-19 (OR: 2.60, 95% CI 1.57 to 4.31, p trend across categories=0.001), longer duration (OR: 2.98, 95% CI 1.10 to 8.05, p trend=0.02) and severity (OR: 3.26, 95% CI 1.25 to 8.48, p trend=0.02) compared with reporting no burnout. These associations remained significant after adjusting for frequency of COVID-19 exposures.

**Conclusions:**

In six countries, longer sleep duration was associated with lower odds of COVID-19, but the association with daytime nap may not be consistent across countries. Greater sleep problems and high level of burnout were robustly associated with greater odds of COVID-19. Sleep and burnout may be risk factors for COVID-19 in high-risk HCWs.

**What this paper adds**

- In 2884 high-risk healthcare workers from six countries, every 1-hour increase in sleep duration at night was associated with 12% lower odds of COVID-19 while, conversely, having severe sleep problems was associated with 88% greater odds of COVID-19.
- Daily burnout from work was associated with 2.6-fold greater odds of COVID-19 as well as greater disease duration and severity.
- Lack of sleep at night, severe sleep problems and high level of burnout from work may be risk factors for COVID-19 among healthcare workers, highlighting the importance of healthcare professionals’ well-being during the pandemic.

## Introduction

Front-line healthcare workers (HCWs) are at high risk of COVID-19 infection, caused by SARS-CoV-2.^1^ During the global pandemic, HCWs have played a vital role in treating patients with COVID-19 and containing community transition of COVID-19. As such, HCWs are particularly vulnerable to contracting the infection, evidenced by a recent Scottish study which found that the risk of hospital admission due to COVID-19 was 1.8-fold to 3.3-fold higher in HCWs treating patients with COVID-19 and their household members compared with HCWs who were not treating patients with COVID-19.^2^

Although exposure to SARS-CoV-2 is the most potent risk factor, it is important to understand if HCWs’ well-being contributes to higher risk of COVID-19 infection. Specifically, sleep habits and stress have been shown to increase risk of infectious diseases. For instance, in 164 healthy men and women 18–55 years of age, short sleep duration was associated with greater odds of developing a cold after viral exposure.^3^ Similarly, a high level of burnout was prospectively associated with a higher risk of common cold and influenza-like illness in the general population.^4^ Only one study examined the association between sleep duration and COVID-19 in 431 Chinese adults and found that lack of sleep was associated with greater severity of COVID-19 compared with the recommended sleep status.^5^ However, this prior study did not have detailed questions on sleep habits, and no previous studies have examined whether other sleep habits and self-reported burnout are associated with COVID-19. Understanding these factors can help inform public health measures to support HCWs during the pandemic.

Thus, we aimed to assess whether sleep habits and self-reported burnout may be risk factors for COVID-19 among at-risk HCWs.

## Methods

### Study design and study population

The present study was a population-based case–control study conducted from 17 July to 25 September 2020. From 1.5 million registered American and European HCWs in the Survey Healthcare Globus (SHG) network, we identified HCWs with high frequency of exposure to patients with COVID-19 based on predetermined medical specialty (eg, critical care, emergency medicine, internal medicine). Sample size (n=3000) was predetermined and was higher for the USA (n=1000) than European countries (n=400 for each country), taking into account a higher number of registered US HCWs in the SHG. We aimed to recruit 3000 participants (n=500 cases; n=2500 controls) to obtain 80% statistical power based on the assumption of a prevalence of prespecified lifestyle exposures (eg, proportion of individuals with high fat intake) of at least 10% in controls, an OR of at least 1.40 and 5% level of significance.^6^

Details on the study design and study population have been reported previously.^7^ Briefly, we focused on HCWs with high frequency of exposure to patients with COVID-19. To enter into the study, all participants must have had significant exposure to patients with COVID-19. To qualify, all participants answered in the affirmative to the question: ‘To your knowledge, have you had significant close contact with COVID-19 (SARS-CoV2) patients in your workplace?’ In order to further define the significance of exposure, participants were asked: ‘On a typical shift during the COVID-19 pandemic, how frequently were you in close proximity to patients or others with COVID-19?’ Participants were ineligible if (1) they had answered that they had infrequent exposure to patients with COVID-19 (defined as <5% of time on a typical shift), unless they either had COVID-19-like illness or tested positive for COVID-19, to suggest significant, close exposure; (2) their medical specialty was not one of the prespecified high-risk specialties; or (3) their practice setting was not considered high risk (eg, private practice). Participants could not complete the questionnaire if their response on disease severity did not match their description of COVID-19 symptoms. There were 7334 participants screened for eligibility, and 2844 HCWs with high frequency of exposure to patients with COVID-19 in France, Germany, Italy, Spain, UK and USA met the eligibility criteria.

We selected case–control design because we expected COVID-19 cases to be rarer than controls. At the time the study was designed, COVID-19 was new; thus the number of confirmed cases was much lower than as of 21 January 2021 (1.8 million cumulative cases in April 2020 compared with 98 million cumulative cases across the world in January 2021).^8^ Cases and controls were defined as participants who entered the study, and the number of cases was much fewer than controls, which was what we expected.

HCWs completed a detailed web-based questionnaire (approximately 100 items) on demographics, prior medical diagnosis, medication use and dietary supplement use, dietary habits, physical activity, sleep habits, and burnout from work. Questionnaires were translated as appropriate for each country. A copy of the questionnaire has been published.^7^ All participants provided informed consent electronically.

### COVID-19 cases, severity, duration and controls

COVID-19 cases were defined as either symptomatic cases, answering in the affirmative to the question ‘Since exposure, have you personally experienced symptoms consistent with a diagnosis of COVID-19 (fever, coughing, fatigue, loss of taste or smell)?’, or asymptomatic cases, a positive COVID-19 PCR or antibody test result without COVID-19-like symptoms (fever, cough, fatigue, loss of taste or smell). In the present study, 568 participants were classified as COVID-19 cases based on these criteria. Controls were defined as having a negative COVID-19 test result and no symptoms consistent with COVID-19 illness. Of the participants, 2316 were classified as controls. We used symptoms to define COVID-19 cases because many HCWs in Europe and the USA did not have adequate and timely access to COVID-19 testing in the early phases of the COVID-19 pandemic, when this study was carried out. We considered symptoms of COVID-19 important in defining COVID-19 cases because absence of testing, testing negative for antibodies, or negative viral PCR if not administered in the proper time frame, does not suggest that an HCW, in fact, did not have COVID-19 illness.

In terms of severity of COVID-19 illness, participants had five options to choose from: (1) very mild: asymptomatic or nearly asymptomatic; (2) mild: fever <38°C (without treatment), with or without cough, no dyspnoea, no gasping and no abnormal imaging findings; (3) moderate: fever, respiratory symptoms and/or imaging findings of pneumonia; (4) severe: meet any of the following: (a) respiratory distress, with respiratory rate ≥30 times per minute, (b) low oxygen saturation <93% at rest and (c) partial pressure of oxygen/fraction of inspired oxygen ≤300 mm Hg; and (5) critical: respiratory failure needing mechanical assistance, intensive care unit admission, shock or extrapulmonary organ failure. No participant reported ‘critical’ severity of COVID-19 illness.

Participants reported the number of days they experienced COVID-19 symptoms by answering an open-ended question: ‘How many days did you experience symptoms of COVID-19? Please answer from the first day that you experienced any symptoms until you were completely asymptomatic’. Asymptomatic individuals with a positive PCR or antibody test were considered to have 0 days of duration of symptoms.

### Sleep habits and stress

Participants reported the average duration of sleep per night and napping during daytime hours over the past year prior to the COVID-19 pandemic. Participants were also asked to report sleep problems prior to the COVID-19 pandemic based on three questions: (1) ‘Did you have difficulties falling asleep at night?’ (2) ‘Did you often wake up in the early hours, unable to get back to sleep?’ (3) ‘Did you take sleeping pills more than 3 times per week?’ Participants could answer either ‘yes’ or ‘no’ to these questions. These three questions have often been used to assess sleep problems in adults in epidemiological studies.^9 10^ We assessed the associations between sleep problems and COVID-19 by examining (1) each question separately, (2) creating a binary variable for the presence of any sleep problem (answering ‘yes’ to any of the three questions) and (3) creating a score of 0–3 based on the number of sleep problems. For each question, participants received a score of 1 if they answered ‘yes’ and received a score of 0 if they answered ‘no’.

Participants rated burnout from work based on the question: ‘Prior to the COVID-19 pandemic, I felt burned out from my work’. Participants had seven options to choose from, ranging from never to every day. This question has been widely used to assess burnout in medical personnel and is strongly correlated with the full Maslach Burnout Inventory.^11 12^ We defined self-reported burnout using four levels: (1) never, (2) less than one or few times a month (classified as ‘rarely’), (3) one to few times a week (classified as ‘weekly’) and (4) every day.

### Statistical analyses

We examined the characteristics of the overall study population by case status using per cent and frequencies for categorical variables and mean and SD for continuous variables. To compare differences, we used χ^2^ test and analysis of variance, as appropriate.

We evaluated the association between sleep, burnout and COVID-19 using three progressively adjusted multivariable logistic regression models. For models on sleep, we adjusted for the following covariates: model 1 adjusted for demographic characteristics (age, sex, race/ethnicity and country); model 2 additionally adjusted for specialty and presence of a medical condition (any of the following conditions: pre-diabetes, diabetes, high cholesterol, hypertension, cancer, coronary disease or heart attack, heart failure, prior lung disease, prior lung infection, asthma, overweight or autoimmune disease) and burnout from work; and model 3 additionally adjusted for potential COVID-19 exposures, including frequency of contact with a patient with COVID-19 at work, close exposure to a patient with COVID-19 inside the workplace without personal protective equipment (PPE) and close exposure to a patient with COVID-19 outside the workplace. For sleep hours and daytime napping, we tested for interaction by country to examine if sleep patterns differ by geographical locations. We did not find a significant interaction (p for interaction ≥0.06), but still stratified by country because the direction of the association differed. Then, we evaluated the association between burnout and COVID-19 using the same set of covariates, except that we did not adjust for burnout from work in model 2. Lastly, we examined the association between sleep habits, burnout from work and duration (>14 days vs ≤14 days) and severity of COVID-19-like illness (moderate-to-severe vs very mild to mild) only among COVID-19 cases.

Although the study was not powered based on testing alone, as a sensitivity analysis we excluded COVID-19 cases which were defined only by symptoms, and repeated the analyses on sleep habits and self-reported burnout and COVID-19. All analyses were conducted using Stata V.15 statistical software.

## Results

The 2884 exposed HCWs were predominantly men, with a mean age of 48 years and diverse race/ethnicity (77% white, 12% Asian, 6% mixed, 2% African, 1% other) (table 1). Nearly one-third of the analytic sample did not get or did not have access to a PCR or antibody test. Compared with controls (n=2316), cases (n=568) were slightly younger, more likely to be physicians and more likely to practise internal medicine. Cases were also more likely to report fewer sleep hours at night, slightly more daytime napping hours and have one or more sleep problems. Cases were also more likely to report burnout from work at least monthly.

**Table 1:** Characteristics of healthcare workers exposed to COVID-19, by case status

	Total N=2884	Controls n=2316	Cases n=568	P value
Gender, n (%)				0.63
Female	794 (27.5)	640 (27.6)	154 (27.1)	
Male	2066 (71.6)	1656 (71.5)	410 (72.2)	
Other	2 (0.1)	1 (<1)	1 (0.2)	
Prefer not to say	22 (0.8)	19 (0.8)	3 (0.5)	
Age, years, mean (SD)	48 (10)	48 (10)	47 (10)	0.11
Country, n (%)				<0.001
USA	1061 (36.8)	901 (38.9)	160 (28.2)	
UK	327 (11.3)	233 (10.1)	94 (16.5)	
Spain	528 (18.3)	382 (16.5)	146 (25.7)	
Italy	433 (15.0)	359 (15.5)	74 (13.0)	
Germany	279 (9.7)	233 (10.1)	46 (8.1)	
France	256 (8.9)	208 (9.0)	48 (8.5)	
Race/ethnicity, n (%)				0.49
White	2218 (77)	1792 (77.4)	426 (75.0)	
Any mixed/multiple ethnic background	162 (6)	121 (5.2)	41 (7.2)	
Asian	336 (12)	271 (11.7)	65 (11.4)	
African	48 (2)	36 (1.6)	12 (2.1)	
Other	36 (1)	29 (1.3)	7 (1.2)	
Prefer not to say	84 (3)	67 (2.9)	17 (3.0)	
Job classification, n (%)				0.048
Physician	2723 (94.8)	2187 (94.4)	548 (96.5)	
Nurse/nurse practitioner/physician assistant	149 (5.2)	129 (5.6)	20 (3.5)	
Physician specialty, n (%)				<0.001
Other	12 (0.4)	10 (0.4)	2 (0.4)	
Allergy and immunology	29 (1.0)	25 (1.1)	4 (0.7)	
Cardiology	281 (9.7)	227 (9.8)	54 (9.5)	
Critical care	282 (9.8)	230 (9.9)	52 (9.2)	
Emergency medicine	603 (20.9)	512 (22.1)	91 (16.0)	
Endocrinology, diabetes and metabolism	98 (3.4)	74 (3.2)	24 (4.2)	
Gastroenterology	94 (3.3)	77 (3.3)	17 (3.0)	
Haematology	112 (3.9)	85 (3.7)	27 (4.8)	
Infectious disease	100 (3.5)	82 (3.5)	18 (3.2)	
Internal medicine	433 (15.0)	322 (13.9)	111 (19.5)	
Nephrology	53 (1.8)	38 (1.6)	15 (2.6)	
Neurology	107 (3.7)	82 (3.5)	25 (4.4)	
Pulmonology	430 (14.9)	354 (15.3)	76 (13.4)	
Rheumatology	101 (3.5)	69 (3.0)	32 (5.6)	
Nurse/Nurse Practitioner/Physician Assistant practice setting, n (%)				0.36
Emergency room	24 (16.1)	22 (17.1)	2 (10.0)	
Intensive care unit	50 (33.6)	45 (34.9)	5 (25.0)	
Other hospital-based department	75 (50.3)	62 (48.1)	13 (65.0)	
Presence of pre-existing medical conditions*	1264 (44)	1002 (43.3)	262 (46.1)	0.22
COVID-19 test (PCR or antibody), n (%)				<0.001
No—I did not get a test	748 (25.9)	695 (30.0)	53 (9.3)	
No—I did not have access to the test	101 (3.5)	69 (2.9)	32 (5.6)	
Yes—I tested negative	1737 (60.2)	1552 (67.0)	185 (32.6)	
Yes—I tested positive	298 (10.3)	0 (0)	298 (52.5)	
Sleep and burnout from work, mean (SD)				
Number of sleeping hours at night	6.7 (1.1)	6.7 (1.1)	6.6 (1.1)	0.001
Number of daytime napping hours	0.9 (1.9)	0.8 (1.8)	1.0 (2.1)	0.082
Sleep disturbance, n (%)				
Difficulty sleeping at night	632 (21.9)	495 (21.4)	137 (24.1)	0.16
Often wake up at early hours and unable to get back to sleep	756 (26.2)	607 (26.2)	149 (26.2)	0.99
Sleeping pill use >3 times/week	170 (5.9)	130 (5.6)	40 (7.0)	0.19
Sleep disturbance score, n (%)				0.017
Self-report of having one sleep problem†	513 (17.8)	429 (18.5)	84 (14.8)	
Self-report of having two sleep problems‡	383 (13.3)	304 (13.1)	79 (13.9)	
Self-report of having three sleep problems§	93 (3.2)	65 (2.8)	28 (4.9)	
Frequency of self-reported burnout, n (%)				0.004
Never	500 (17.3)	422 (18.2)	78 (13.7)	
<1 or few times a month (rarely)	1815 (62.9)	1444 (62.4)	371 (65.4)	
1 to few times a week (weekly)	467 (16.2)	379 (16.3)	88 (15.5)	
Every day	102 (3.5)	71 (3.1)	31 (5.5)	

Values are n (%) for categorical variables and mean (SD) for continuous variables.

Cases are defined as individuals with self-reported COVID-19-like illness (fever, cough, fatigue, loss of taste or smell) or a positive PCR or antibody test.

*Pre-existing medical condition included pre-diabetes, diabetes, high cholesterol, hypertension, cancer, coronary disease or heart attack, heart failure, prior lung disease, prior lung infection, asthma, overweight or autoimmune disease.

†Sleep disturbance was defined as ‘yes’ response to any of the following questions: (1) Do you have difficulties falling asleep at night prior to the COVID-19 pandemic? (2) Do you often wake up in the early hours, unable to get back to sleep prior to the COVID-19 pandemic? (3) Do you take sleeping pills more than three times per week prior to the COVID-19 pandemic?

‡Having two sleep problems was defined as saying yes to two of the three questions.

§Having three sleep problems was defined as saying yes to all of the questions.

After adjusting for demographic characteristics, medical specialty, presence of a medical condition and self-reported burnout, 1-hour longer sleep duration at night was associated with lower odds of COVID-19 (OR: 0.88, 95% CI 0.81 to 0.96), whereas 1-hour higher in daytime napping hours was associated with greater odds of COVID-19 (OR: 1.06, 95% CI 1.01 to 1.12) (table 2). Having three sleep problems (difficulty sleeping at night, poor sleep continuity, frequent sleeping pill use) compared with having no sleep problems was associated with almost twofold greater odds (OR: 1.88, 95% CI 1.17 to 3.01, p trend=0.22) of COVID-19, although there was no significant trend across the number of sleep problems. All of these associations remained significant even after adjusting for frequency of COVID-19 exposures. Individual sleep problem components and presence of a sleep problem were not associated with COVID-19.

**Table 2:** Adjusted OR and 95% CI for the association between sleep and COVID-19* (N=2884)

	OR (95% CI)								
	Model 1†	P value	P trend	Model 2‡	P value	P trend	Model 3§	P value	P trend
Number of sleeping hours at night	**0.87 (0.80 to 0.95)**	**0.001**	–	**0.88 (0.81 to 0.96)**	**0.003**	–	**0.89 (0.81 to 0.97)**	**0.003**	–
Number of daytime napping hours	**1.06 (1.01 to 1.12)**	**0.02**	–	**1.06 (1.01 to 1.12)**	**0.02**	–	**1.06 (1.01 to 1.11)**	**0.03**	–
Sleep disturbance									
Difficulty sleeping at night (yes)	1.22 (0.98 to 1.52)	0.08	–	1.15 (0.92 to 1.45)	0.22	–	1.13 (0.90 to 1.42)	0.29	–
Often wake up at early hours and unable to get back to sleep (yes)	1.09 (0.88 to 1.35)	0.41	–	1.04 (0.83 to 1.29)	0.73	–	0.99 (0.79 to 1.23)	0.94	–
Sleeping pill use >3 times/week (yes)	1.42 (0.98 to 2.07)	0.06	–	1.35 (0.92 to 1.96)	0.12	–	1.27 (0.87 to 1.86)	0.22	–
Self-reported sleep disturbance score¶									
No sleep problem	Ref	–	0.07	Ref	–	0.22	Ref	–	0.40
Self-report of having one sleep problem**	0.85 (0.65 to 1.10)	0.21	–	0.82 (0.63 to 1.07)	0.14	–	0.79 (0.60 to 1.03)	0.08	–
Self-report of having two sleep problems††	1.11 (0.84 to 1.46)	0.46	–	1.04 (0.78 to 1.38)	0.79	–	1.01 (0.76 to 1.35)	0.95	–
Self-report of having three sleep problems‡‡	**2.03 (1.28 to 3.24)**	**0.003**	–	**1.88 (1.17 to 3.01)**	**0.01**	–	**1.72 (1.07 to 2.78)**	**0.02**	–
Any sleep disturbance (≥1 sleep problem)	1.04 (0.85 to 1.26)	0.72	–	0.98 (0.8 to 1.20)	0.85	–	0.94 (0.77 to 1.16)	0.57	–

Bold font denotes statistically significant associations.

*COVID-19 cases are defined as individuals with self-reported COVID-19-like illness (fever, cough, fatigue, loss of taste or smell) or a positive PCR or antibody test.

†Model 1 adjusted for age, sex, race and country.

‡Model 2 additionally adjusted for specialty, presence of a medical condition and stress from work (feeling burned out).

§Model 3 additionally adjusted for frequency of contact with a patient with COVID-19 at work, close exposure to a patient with COVID-19 inside the workplace without personal protective equipment and close exposure outside the workplace.

¶Sleep disturbance was defined as “yes” response to any of the following questions: a) Do you have difficulties falling asleep at night prior to the Covid-19 pandemic?, b) Do you often wake up in the early hours, unable to get back to sleep prior to the Covid-19 pandemic?, or c) Do you take sleeping pills more than 3 times per week prior to the Covid-19 pandemic?

**Having 1 sleep problem was defined as saying 'yes' to any of the three questions.

††Having 2 sleep problems was defined as saying yes to two of the three questions.

‡‡Having 3 sleep problems was defined as saying yes to all of the three questions.

Ref, reference.

**Figure 1:**
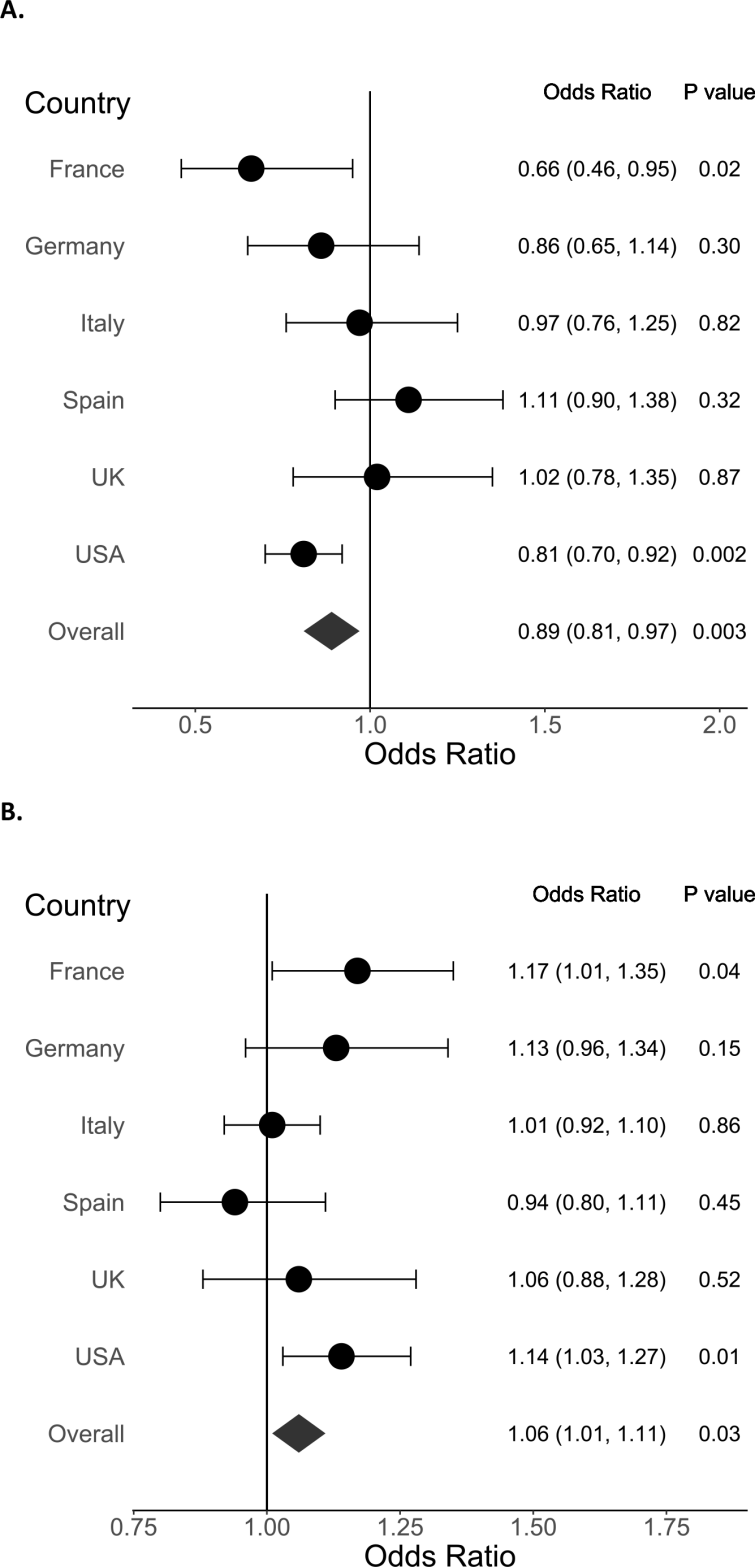
Adjusted OR and 95% CI for the association between (A) sleeping hours at night and (B) daytime napping hours and COVID-19 (N=2884). ORs are calculated for each country adjusting for age, sex, race, specialty, presence of a medical condition, burnout from work, frequency of contact with a patient with COVID-19 at work, close exposure to a patient with COVID-19 inside the workplace without personal protective equipment and close exposure outside the workplace.

When we stratified sleeping hours at night and daytime napping hours by country, the associations were statistically significant in the fully adjusted models for France and USA, with patterns consistent with the overall study population (figure 1A,B). In Spain, daytime napping hours was associated with a slightly lower odds of COVID-19, but the association was not statistically significant (figure 1B).

**Figure 2:**
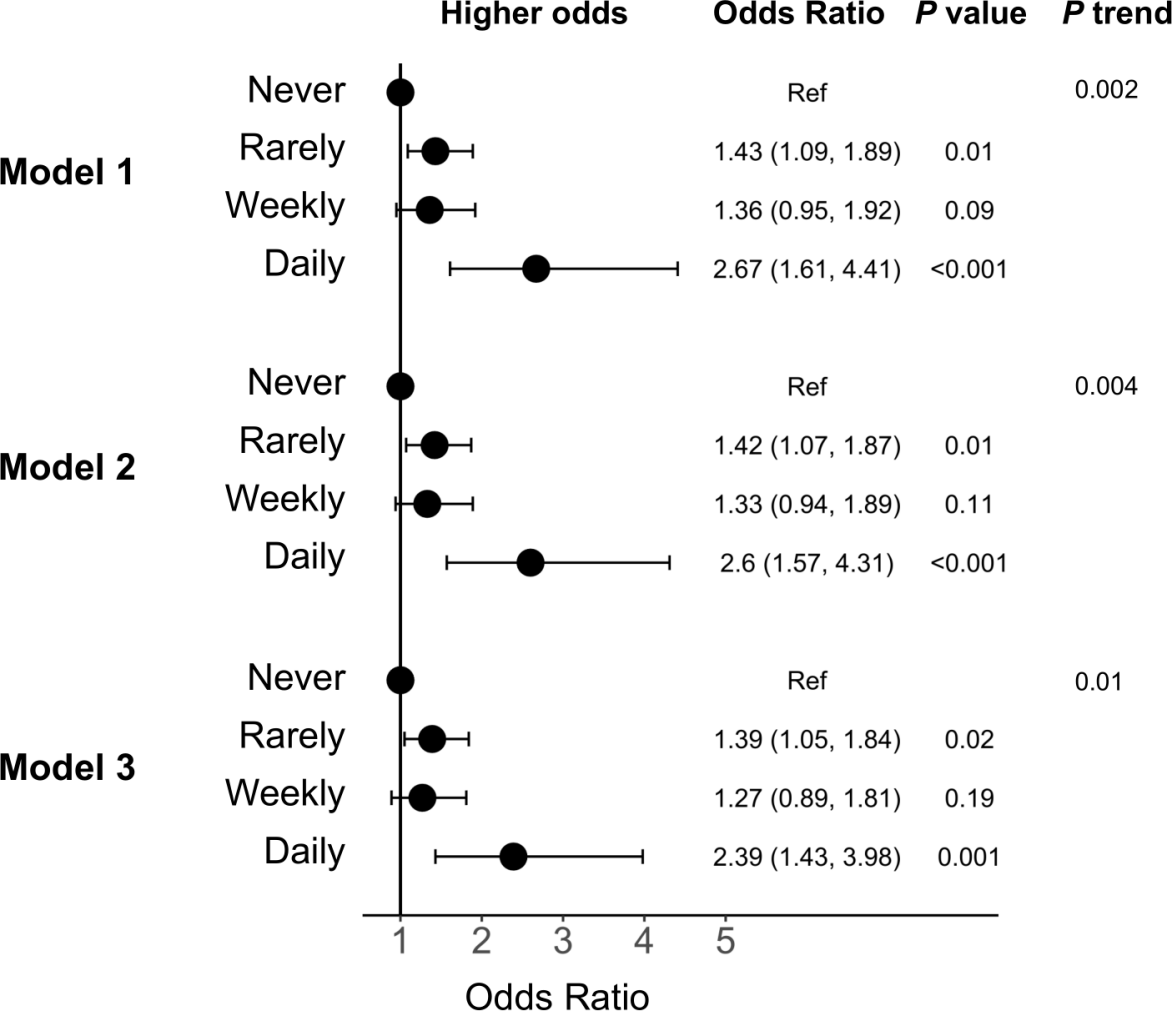
Adjusted OR and 95% CI for the association between burnout from work and COVID-19 (N=2884). Model 1 adjusted for age, sex, race and country. Model 2 additionally adjusted for specialty and presence of a medical condition. Model 3 additionally adjusted for frequency of contact with a patient with COVID-19 at work, close exposure to a patient with COVID-19 inside the workplace without personal protective equipment and close exposure outside the workplace. Ref, reference.

There was a significant dose–response relationship between frequency of burnout and COVID-19 (p trend ≤0.01 across frequency categories; figure 2). Reporting burnout ‘rarely’ or ‘weekly’ was associated with approximately 1.3-fold to 1.4-fold greater odds of reporting COVID-19 compared with reporting no burnout in the model which adjusted for demographic characteristics, specialty and presence of a medical condition (model 2 rarely vs never OR: 1.42, 95% CI 1.07 to 1.87; weekly vs never OR: 1.33, 95% CI 0.94 to 1.89). Further, reporting burnout ‘every day’ was associated with 2.6-fold greater odds (model 2 OR: 2.60, 95% CI 1.57 to 4.31, p<0.001) of COVID-19 and remained significant after additionally adjusting for COVID-19 exposures (model 3 OR: 2.39, 95% CI 1.43 to 3.98, p=0.001). The magnitude of the association for reporting burnout ‘rarely’ and ‘weekly’ with COVID-19 was similar, but the estimates were less precise for reporting burnout ‘weekly’ compared with ‘rarely’ due to fewer participants reporting weekly burnout.

In the fully adjusted models, longer duration of sleep at night was associated with lower odds (OR: 0.83, 95% CI 0.70 to 0.99) of moderate-to-severe COVID-19-like illness (online supplemental table 1). High level of burnout was associated with longer duration of COVID-19-like illness (burnout every day vs never OR: 2.98, 95% CI 1.10 to 8.05, p trend=0.02) and severity (OR: 3.26, 95% CI 1.25 to 8.48, p trend=0.02).

10.1136/bmjnph-2021-000228.supp1 Supplementary data

When we restricted our analyses to a positive PCR or antibody test (n=298 cases), no substantial change was observed for the association between self-reported burnout and COVID-19 in the fully adjusted model (burnout every day vs never OR: 2.60, 95% CI 1.34 to 5.05, p trend=0.04) (online supplemental table 2). The direction and magnitude of the associations between sleep duration at night (OR for 1-hour longer sleep duration at night: 0.91, 95% CI 0.81 to 1.02, p=0.12), sleep problems (OR for having three sleep problems vs none: 1.50, 95% CI 0.79 to 2.85, p trend=0.24) and COVID-19 were similar with the main analysis, although the estimates were not statistically significant due to fewer number of cases. The association between daytime napping hours (OR for 1-hour longer daytime napping: 1.00, 95% CI 0.93 to 1.07, p=0.98) and COVID-19 attenuated.

## Discussion

In exposed HCWs from six countries, longer sleep duration at night was associated with lower odds of COVID-19. In contrast, greater number of daytime napping hours, reporting three sleep problems and higher level of burnout were robustly associated with greater odds of COVID-19. However, the association between daytime napping hours and COVID-19 differed by country. High level of burnout was associated with longer duration and severity of COVID-19-like illness. Our findings add to the literature that sleep duration at night, sleep problems and burnout may be risk factors for viral illnesses like COVID-19.

To our knowledge, this study is the first to report an association between detailed sleep habits (sleep hours at night, daytime napping hours, severe sleep problems) and COVID-19 in multiple countries. It has been speculated that insufficient sleep may play an important role in COVID-19.^13 14^ One prior study found that self-reported lack of sleep was associated with higher odds whereas 1-hour longer daily sleep time was associated with lower odds of ≥20 days of hospital stay due to COVID-19.^5^ Our results on sleep hours at night are in agreement with this previous study, but we contribute to the literature by providing more comprehensive information on sleep habits. Several prior studies have already shown that short sleep duration prior to virus exposure is prospectively associated with a greater risk of acute infectious diseases, such as pneumonia and common cold.^3 15 16^ Similarly, self-reported sleep disorder has been associated with greater odds of head or chest cold.^17^ The mechanism underlying these associations remains unclear, but it has been hypothesized that lack of sleep and sleep disorders may adversely influence the immune system by increasing proinflammatory cytokines and histamines.^3 15^ However, it is important to note that our study used self-reported sleep duration and self-reported sleep disorders, and the association between daytime napping and COVID-19 attenuated when we restricted COVID-19 cases to a positive PCR or antibody test. Future studies are needed to confirm whether these associations persist with objective measures of sleep.

Similar to sleep, burnout has been reported to be cross-sectionally associated with the common cold and influenza-like illnesses and prospectively associated with influenza-like illnesses in the general population.^4^ Burnout has also been associated with chronic diseases, such as diabetes, cardiovascular disease, musculoskeletal disease and all-cause mortality.^18–21^ These studies have suggested that burnout may directly or indirectly predict illnesses by (1) occupational stress impairing the immune system and changing cortisol levels, and (2) occupational stress increasing risky health behaviours.^4 20^ In the context of the global pandemic, it is unlikely that burnout increases risky behaviours (eg, not wearing PPE) among healthcare professionals, which would then increase virus exposure. Considering the reported associations in higher workload and burnout,^22^ it is possible that individuals reporting burnout ‘every day’ cared for more patients with COVID-19 than those who reported no burnout, which may have increased exposure to coronavirus. Another consideration may be that burnout leads to fatigue and increased errors in donning and doffing PPE or hand hygiene lapses, thereby increasing risk. However, even when we adjusted for COVID-19 exposures, the association between burnout and COVID-19 persisted, suggesting that a negative impact on immune system may have played an important role.

We found the association between daytime napping hours and odds of COVID-19 interesting. There may be cultural differences in daytime napping. Daytime napping may be a marker of sleep deprivation or stress in some countries, while it may be a tradition (eg, siesta or afternoon nap) in Spain.^23^ In line with this hypothesis, when we stratified the association between daytime napping and COVID-19 by country, there was a non-significant inverse association for Spain and a positive association for other countries (eg, France and USA). We did not ask why participants took daytime naps; thus we could not further explore this association. Daytime napping appears to have variable association by country, which warrants further investigation.

The strengths of our study include the inclusion of high-risk HCWs from six different countries at the height of the pandemic, use of questions on sleep problems and burnout from work that have been widely tested in epidemiological studies, and rigorous adjustment of covariates. We were able to identify at-risk HCWs in the early phase of the pandemic by leveraging a large network of HCWs. However, several limitations should be noted. Our sampling was restricted to HCWs who were registered in the SHG network. Although this database has 1.5 million registered HCWs, our findings may not be generalisable to HCWs who are not in this database. Participants also self-reported their exposure, outcome and covariates. We cannot exclude the possibility of recall bias, which may have led to inaccurate reports on sleep hours and daytime napping hours. In our questionnaire, we did not define a time frame of the COVID-19 pandemic; thus it is possible that participants may have incorrectly referenced their sleep habits and burnout. However, HCWs are likely a reliable source of information. Next, asymptomatic individuals with a positive PCR or antibody test were considered to have 0 days of COVID-19 symptom duration. This may not be precise, but it is unclear how duration of COVID-19 illness can be estimated among asymptomatic individuals. Our sampling might also have captured only very mild to moderate severity of COVID-19 cases because participants with critical cases (respiratory failure, admission to intensive care unit or extrapulmonary organ failure) may have not been able to complete the questionnaire. Lastly, although we adjusted for a wide range of covariates, there may still be residual confounding factors.

In conclusion, we found that lack of sleep at night, severe sleep problems and high level of burnout may be risk factors for COVID-19 in front-line HCWs. The association between daytime napping and COVID-19 appears to differ between Spain and the rest of the countries. Our results highlight the importance of healthcare professionals’ well-being during the pandemic. Awareness of these risk factors in HCWs will be helpful in maintaining a healthy and productive workforce.
