# Supplementary material for: COVID-19 illness in relation to sleep and burnout
Source: BMJ Nutr Prev Health. 2021 Mar 22;4(1):132–9. doi: 10.1136/bmjnph-2021-000228 (PMC7995669; doi:10.1136/bmjnph-2021-000228)
Supplement: Supplementary data [file bmjnph-2021-000228supp001.pdf]

Title: Covid-19 illness in relation to sleep and burnout

Hyunju Kim PhD, Sheila Hegde MD, Christine LaFiura BA, Madhunika Raghavan MA, Eric Luong MPH, Susan Cheng MD, Casey M Rebholz PhD, Sara B Seidelmann MD PhD

Supplemental Table 1. Adjusted Odds Ratios (OR) and 95% Confidence Intervals (CI) for the association between Sleep and Duration and Severity of Covid-19 like illness (n=568)[a]

Supplemental Table 2. Adjusted Odds Ratios (OR) and 95% Confidence Intervals (CI) for the Association between Sleep and Covid-19 test (PCR or antibody)[a]

Supplemental Table 1. Adjusted Odds Ratios (OR) and 95% Confidence Intervals (CI) for the association between Sleep and Duration and Severity of Covid-19 like illness (n=568)[a]

	**> 14 days of symptoms[b]**			**Moderate to severe symptoms[c]**		
	OR (95% CI)	*P*-value	*P*-trend	OR (95% CI)	*P*-value	*P*-trend
**Number of sleeping hours at night**	0.92 (0.76, 1.12)	0.42	–	**0.83 (0.70, 0.99)**	**0.03**	–
**Number of daytime napping hours**	0.93 (0.82, 1.05)	0.25	–	1.06 (0.97, 1.17)	0.21	–
**Sleep disturbance**						
Difficulty sleeping at night (*yes*)	1.13 (0.68, 1.88)	0.63	–	1.04 (0.65, 1.67)	0.87	–
Often wake up at early hours and unable to get back to sleep (*yes*)	0.98 (0.60, 1.60)	0.93	–	0.86 (0.54, 1.37)	0.53	–
Sleeping pill use >3 times/wk (*yes*)	1.35 (0.59, 3.07)	0.48	–	1.28 (0.6, 2.72)	0.52	–
**Self-reported sleep disturbance score**						
No sleep problem						
Self-report of having 1 sleep problem	0.90 (0.49, 1.67)	0.74	–	0.59 (0.32, 1.11)	0.10	–
Self-report of having 2 sleep problems	1.21 (0.66, 2.22)	0.54	–	1.06 (0.6, 1.88)	0.84	–
Self-report of having 3 sleep problems	1.05 (0.37, 2.94)	0.93	–	1.09 (0.44, 2.71)	0.85	–
**Any sleep disturbance (≥1 sleep problem)**	1.04 (0.66, 1.65)	0.86	–	0.84 (0.54, 1.29)	0.43	–
**Frequency of self-reported burnout**			**0.02**			**0.01**
Never						
Rarely	0.93 (0.47, 1.82)	0.83	–	1.00 (0.55, 1.83)	0.99	–
Weekly	1.35 (0.59, 3.06)	0.47	–	1.47 (0.69, 3.12)	0.32	–
Every day	**2.98 (1.10, 8.05)**	**0.03**	–	**3.26 (1.25, 8.48)**	**0.02**	–

[a] adjusted for age, sex, race, country, specialty, presence of a medical condition, stress from work (adjusted only for sleep variables), frequency of contact with a Covid-19 patient at work, close exposure to a Covid-19 patient inside the workplace without PPE and close exposure outside of the workplace

[b] Participants reported the number of days they experienced symptoms of Covid-19. Asymptomatic individuals with a positive PCR or antibody test were considered to have 0 days of Covid-19 symptom duration.

[c] Moderate to severe severity was compared to very mild to mild severity. 'Very mild' severity was defined as asymptomatic or nearly asymptomatic. 'Mild' severity was defined as symptoms [fever <38°C (without treatment), with or without cough, no dyspnea, no gasping, no abnormal imaging findings]. 'Moderate' severity was defined as fever, respiratory symptoms, and/or imaging findings of pneumonia. 'Severe'

severity was defined as meet any of the following: 1) respiratory distress, Respiratory Rate ≥30 times/min 2) low oxygen saturation (SpO2) <93% at rest 3) partial pressure of oxygen (PaO2)/ fraction of inspired oxygen (FiO2) ≤ 300 mm Hg.

Supplemental Table 2. Adjusted Odds Ratios (OR) and 95% Confidence Intervals (CI) for the Association between Sleep and Covid-19 test (PCR or antibody)[a]

	OR (95% CI)								
	**Model 1[b]**	***P*-value**	***P*-trend**	**Model 2[c]**	***P*-value**	***P*-trend**	**Model 3[d]**	***P*-value**	***P*-trend**
**Number of sleeping hours at night**	0.89 (0.80, 1.01)	0.08	--	0.91 (0.81, 1.01)	0.09	--	0.91 (0.81, 1.02)	0.12	--
**Number of daytime napping hours**	1.00 (0.93, 1.07)	0.96	--	1.00 (0.93, 1.07)	0.99	--	1.00 (0.93, 1.07)	0.98	--
**Sleep disturbance**									
Difficulty sleeping at night (*yes*)	0.93 (0.67, 1.26)	0.63	--	0.88 (0.64, 1.21)	0.43	--	0.86 (0.62, 1.19)	0.36	--
Often wake up at early hours and unable to get back to sleep (*yes*)	0.85 (0.63, 1.15)	0.30	--	0.81 (0.59, 1.10)	0.17	--	0.77 (0.56, 1.05)	0.10	--
Sleeping pill use >3 times/week (*yes*)	1.25 (0.75, 2.12)	0.39	--	1.21 (0.72, 2.05)	0.47	--	1.15 (0.68, 1.95)	0.60	--
**Self-reported sleep disturbance score**									
No sleep problem	Ref	--	0.95	Ref	--	0.34	Ref	--	0.24
Self-report of having 1 sleep problem	0.81 (0.57, 1.14)	0.23	--	0.78 (0.55, 1.10)	0.17	--	0.75 (0.53, 1.07)	0.11	--
Self-report of having 2 sleep problems	0.70 (0.46, 1.07)	0.10	--	0.65 (0.42, 1.00)	0.05	--	**0.63 (0.41, 0.97)**	**0.04**	--
Self-report of having 3 sleep problems	1.77 (0.94, 3.33)	0.07	--	1.64 (0.87, 3.11)	0.12	--	1.50 (0.79, 2.85)	0.21	--
**Any sleep disturbance (≥1 sleep problem)**	0.84 (0.64, 1.09)	0.19	--	0.79 (0.60, 1.04)	0.11	--	0.76 (0.58, 1.01)	0.06	--
**Frequency of self-reported burnout**									
Never	Ref	--	**0.02**	Ref	--	**0.02**	Ref	--	**0.04**
Rarely	1.46 (1.00, 2.12)	0.05		1.46 (1.00, 2.13)	0.05		1.45 (0.99, 2.10)	0.05	
Weekly	1.36 (0.85, 2.17)	0.19		1.36 (0.85, 2.17)	0.25		1.30 (0.82, 2.09)	0.32	
Every day	**2.80 (1.45, 5.39)**	**0.009**		**2.71 (1.46, 5.43)**	**0.002**		**2.60 (1.34, 5.05)**	**0.005**	

[a] Covid-19 cases are defined as individuals with positive PCR or antibody test (N=298 cases and N=2316 controls).
[b] Model 1 adjusted for age, sex, race, and country.
[c] Model 2 additionally adjusted for specialty, presence of a medical condition, and stress from work (feeling burned out; only adjusted for sleep habits).
[d] Model 3 additionally adjusted for frequency of contact with a Covid-19 patient at work, close exposure to a Covid-19 patient inside the workplace without PPE and close exposure outside of the workplace.

Bold font denotes statistically significant associations.
