# Supplementary material for: COVID-19 illness in relation to sleep and burnout
Source: BMJ Nutr Prev Health. 2021 Mar 22:bmjnph-2021-000228. doi: 10.1136/bmjnph-2021-000228 (PMC7995669; doi:10.1136/bmjnph-2021-000228)
Supplement: Supplementary file 1 [file bmjnph-2021-000228supp001.pdf]

Title: Covid-19 illness in relation to sleep and burnout

Hyunju Kim PhD, Sheila Hegde MD, Christine LaFiura BA, Madhunika Raghavan MA, Eric Luong MPH, Susan Cheng MD, Casey M Rebholz PhD, Sara B Seidelmann MD PhD

Supplemental Table 1. Adjusted Odds Ratios (OR) and 95% Confidence Intervals (CI) for the association between Sleep and Duration and Severity of Covid-19 like illness (n=568)[a]

Supplemental Table 2. Adjusted Odds Ratios (OR) and 95% Confidence Intervals (CI) for the Association between Sleep and Covid-19 test (PCR or antibody)[a]

Supplemental Table 1. Adjusted Odds Ratios (OR) and 95% Confidence Intervals (CI) for the association between Sleep and Duration and Severity of Covid-19 like illness (n=568)[a]

	**> 14 days of symptoms[b]**			**Moderate to severe symptoms[c]**		
	OR (95% CI)	*P*-value	*P*-trend	OR (95% CI)	*P*-value	*P*-trend
**Number of sleeping hours at night**	0.92 (0.76, 1.12)	0.42	–	**0.83 (0.70, 0.99)**	**0.03**	–
**Number of daytime napping hours**	0.93 (0.82, 1.05)	0.25	–	1.06 (0.97, 1.17)	0.21	–
**Sleep disturbance**						
Difficulty sleeping at night (*yes*)	1.13 (0.68, 1.88)	0.63	–	1.04 (0.65, 1.67)	0.87	–
Often wake up at early hours and unable to get back to sleep (*yes*)	0.98 (0.60, 1.60)	0.93	–	0.86 (0.54, 1.37)	0.53	–
Sleeping pill use >3 times/wk (*yes*)	1.35 (0.59, 3.07)	0.48	–	1.28 (0.6, 2.72)	0.52	–
**Self-reported sleep disturbance score**						
No sleep problem						
Self-report of having 1 sleep problem	0.90 (0.49, 1.67)	0.74	–	0.59 (0.32, 1.11)	0.10	–
Self-report of having 2 sleep problems	1.21 (0.66, 2.22)	0.54	–	1.06 (0.6, 1.88)	0.84	–
Self-report of having 3 sleep problems	1.05 (0.37, 2.94)	0.93	–	1.09 (0.44, 2.71)	0.85	–
**Any sleep disturbance (≥1 sleep problem)**	1.04 (0.66, 1.65)	0.86	–	0.84 (0.54, 1.29)	0.43	–
**Frequency of self-reported burnout**			**0.02**			**0.01**
Never						
Rarely	0.93 (0.47, 1.82)	0.83	–	1.00 (0.55, 1.83)	0.99	–
Weekly	1.35 (0.59, 3.06)	0.47	–	1.47 (0.69, 3.12)	0.32	–
Every day	**2.98 (1.10, 8.05)**	**0.03**	–	**3.26 (1.25, 8.48)**	**0.02**	–

[a] adjusted for age, sex, race, country, specialty, presence of a medical condition, stress from work (adjusted only for sleep variables), frequency of contact with a Covid-19 patient at work, close exposure to a Covid-19 patient inside the workplace without PPE and close exposure outside of the workplace

[b] Participants reported the number of days they experienced symptoms of Covid-19. Asymptomatic individuals with a positive PCR or antibody test were considered to have 0 days of Covid-19 symptom duration.

[c] Moderate to severe severity was compared to very mild to mild severity. 'Very mild' severity was defined as asymptomatic or nearly asymptomatic. 'Mild' severity was defined as symptoms [fever <38°C (without treatment), with or without cough, no dyspnea, no gasping, no abnormal imaging findings]. 'Moderate' severity was defined as fever, respiratory symptoms, and/or imaging findings of pneumonia. 'Severe'

severity was defined as meet any of the following: 1) respiratory distress, Respiratory Rate ≥30 times/min 2) low oxygen saturation (SpO2) <93% at rest 3) partial pressure of oxygen (PaO2)/ fraction of inspired oxygen (FiO2) ≤ 300 mm Hg.

BMJ Publishing Group Limited (BMJ) disclaims all liability and responsibility arising from any reliance placed on this supplemental material which has been supplied by the author(s)

Supplemental Table 2. Adjusted Odds Ratios (OR) and 95% Confidence Intervals (CI) for the Association between Sleep and Covid-19 test (PCR or antibody)[a]

	**OR (95% CI)**								
	**Model 1[b]**	***P*-value**	***P*-trend**	**Model 2[c]**	***P*-value**	***P*-trend**	**Model 3[d]**	***P*-value**	***P*-trend**
**Number of sleeping hours at night**	0.89 (0.80, 1.01)	0.08	--	0.91 (0.81, 1.01)	0.09	--	0.91 (0.81, 1.02)	0.12	--
**Number of daytime napping hours**	1.00 (0.93, 1.07)	0.96	--	1.00 (0.93, 1.07)	0.99	--	1.00 (0.93, 1.07)	0.98	--
**Sleep disturbance**									
Difficulty sleeping at night (*yes*)	0.93 (0.67, 1.26)	0.63	--	0.88 (0.64, 1.21)	0.43	--	0.86 (0.62, 1.19)	0.36	--
Often wake up at early hours and unable to get back to sleep (*yes*)	0.85 (0.63, 1.15)	0.30	--	0.81 (0.59, 1.10)	0.17	--	0.77 (0.56, 1.05)	0.10	--
Sleeping pill use >3 times/week (*yes*)	1.25 (0.75, 2.12)	0.39	--	1.21 (0.72, 2.05)	0.47	--	1.15 (0.68, 1.95)	0.60	--
**Self-reported sleep disturbance score**									
No sleep problem	Ref	--	0.95	Ref	--	0.34	Ref	--	0.24
Self-report of having 1 sleep problem	0.81 (0.57, 1.14)	0.23	--	0.78 (0.55, 1.10)	0.17	--	0.75 (0.53, 1.07)	0.11	--
Self-report of having 2 sleep problems	0.70 (0.46, 1.07)	0.10	--	0.65 (0.42, 1.00)	0.05	--	**0.63 (0.41, 0.97)**	**0.04**	--
Self-report of having 3 sleep problems	1.77 (0.94, 3.33)	0.07	--	1.64 (0.87, 3.11)	0.12	--	1.50 (0.79, 2.85)	0.21	--
**Any sleep disturbance (≥1 sleep problem)**	0.84 (0.64, 1.09)	0.19	--	0.79 (0.60, 1.04)	0.11	--	0.76 (0.58, 1.01)	0.06	--
**Frequency of self-reported burnout**									
Never	Ref	--	**0.02**	Ref	--	**0.02**	Ref	--	**0.04**
Rarely	1.46 (1.00, 2.12)	0.05		1.46 (1.00, 2.13)	0.05		1.45 (0.99, 2.10)	0.05	
Weekly	1.36 (0.85, 2.17)	0.19		1.36 (0.85, 2.17)	0.25		1.30 (0.82, 2.09)	0.32	
Every day	**2.80 (1.45, 5.39)**	**0.009**		**2.71 (1.46, 5.43)**	**0.002**		**2.60 (1.34, 5.05)**	**0.005**	

[a] Covid-19 cases are defined as individuals with positive PCR or antibody test (N=298 cases and N=2316 controls).
[b] Model 1 adjusted for age, sex, race, and country.
[c] Model 2 additionally adjusted for specialty, presence of a medical condition, and stress from work (feeling burned out; only adjusted for sleep habits).
[d] Model 3 additionally adjusted for frequency of contact with a Covid-19 patient at work, close exposure to a Covid-19 patient inside the workplace without PPE and close exposure outside of the workplace.

Bold font denotes statistically significant associations.
